# Effects of spinach nitrate on insulin resistance, endothelial dysfunction markers and inflammation in mice with high-fat and high-fructose consumption

**DOI:** 10.3402/fnr.v60.32010

**Published:** 2016-09-09

**Authors:** Ting Li, Xinshan Lu, Yanfei Sun, Xingbin Yang

**Affiliations:** 1 Shaanxi Engineering Laboratory for Food Green Processing and Safety Control, College of Food Engineering and Nutritional Science, Shaanxi Normal University, Xi'an, China; 2 Centre for Cancer and Inflammation Research, School of Chinese Medicine, Hong Kong Baptist University, Hong Kong, China

**Keywords:** spinach, nitrate, dyslipidemia, endothelial function, inflammation, NO

## Abstract

**Background:**

Insulin resistance, which is associated with an increased risk of cardiovascular morbidity and mortality, has become a leading nutrition problem. Inorganic nitrate enriched in spinach has been demonstrated to reverse the pathological features of insulin resistance and endothelial dysfunction. However, the effects of a direct intake of nitrate-enriched spinach on insulin resistance and endothelial dysfunction have not been studied.

**Objective:**

To investigate the effects of spinach nitrate on insulin resistance, lipid metabolism, endothelial function, and inflammation in mice fed with a high-fat and high-fructose diet.

**Design:**

A diet intervention of spinach with or without nitrate was performed in mice. A high-fat and high-fructose diet was used to cause insulin resistance, endothelial dysfunction, and inflammation in mice. The impacts of spinach nitrate on lipid profile, insulin resistance, markers of endothelial function, and inflammation were determined in mice.

**Results:**

Spinach nitrate improved the vascular endothelial function of the mice with high-fat and high-fructose consumption, as evidenced by the elevated plasma nitrite level, increased serum nitric oxide (NO) level and decreased serum ET-1 level after spinach nitrate intervention. Spinach nitrate also reduced serum triglycerides, total cholesterol, and low-density lipoprotein-cholesterol levels and elevated serum high-density lipoprotein-cholesterol levels in the mice fed with a high-fat and high-fructose diet. Mice receiving spinach with 60 mg/kg of nitrate (1.02±0.34) showed a significantly low homeostasis model assessment-insulin resistance index as compared with the model mice (2.05±0.58), which is indicating that spinach nitrate could effectively improve the insulin resistance. In addition, spinach nitrate remarkably decreased the elevated serum C-reactive protein, tumor necrosis factor α, and interleukin-6 levels induced by a high-fat and high-fructose diet.

**Conclusions:**

The intake of spinach nitrate can augment NO status, improve lipid homeostasis, relieve inflammation, and enhance endothelial function, suggesting that spinach is promising dietary supplements for insulin resistance prevention.

Insulin resistance is one of the powerful risk factors for the development of cardiovascular disease (CVD) and type 2 diabetes (1). The prevalence of insulin resistance is increasing, especially in developing countries and in younger populations, with approximately 20 to 40% of prevalence in different populations (2, 3). Increasing evidences support that the presence of insulin resistance, characterized as impaired insulin-stimulated glucose and/or lipid metabolism, represents early events in individuals at high risk for developing CVD (4). Insulin resistance was shown to be associated with increased production of pro-inflammatory cytokines, which are predictors of cardiovascular events (5). Insulin resistance is also linked to endothelial dysfunction in that hyperinsulinemia causes the release of the potent vasoconstrictor endothelin (6). Previous studies have demonstrated that pro-oxidative and pro-inflammatory processes play a pivotal role in endothelial dysfunction (4, 7). Vascular damage resulting from lipid deposition and oxidative stress to the vessel wall triggers an inflammatory reaction, which can worsen the insulin resistance and endothelial dysfunction (4).

Diet exerts important long-term effects on body functions and also plays a vital role in body health and disease development. Indeed, compelling epidemiological evidence suggests that increasing consumption of vegetables reduces the risk of CVD, and the potential cardiovascular benefits of vegetables may be attributed to the high content of inorganic nitrate (8, 9). Several clinical and experimental studies have reported that oral consumption of inorganic nitrate can reduce blood pressure, protect against ischemia–reperfusion injury, prevent endothelial dysfunction, and reverse pathological features of metabolic syndrome including visceral fat accumulation and elevated triglyceride levels, thereby preventing the occurrence and development of CVD (10–13). In addition, dietary nitrate is thought to enhance the production of bioactive nitric oxide (NO) in the body, which is a critical regulator of vascular homeostasis (9). Green leafy vegetables, such as spinach, are among the richest sources of nitrate in the diet and account for approximately 80–85% of the average daily dietary nitrate exposure (14, 15). Although there are some investigations about the protective effects of dietary intake of inorganic nitrate on insulin response and endothelial function, few studies report the influences of a direct intake of nitrate-enriched vegetables on insulin resistance in mice. Therefore, the present study for the first time established a diet intervention method using spinach with or without nitrate to evaluate the impacts of different contents of spinach nitrate on insulin resistance, endothelial dysfunction, and inflammation in mice. More specifically, the effects of spinach nitrate on the lipid metabolism, lipid peroxidation, insulin resistance, endothelial dysfunction markers, and inflammation were evaluated *in vivo*.

## Materials and methods

### Materials and chemicals

The fresh spinach, which was harvested from the greenhouse in winter, was purchased from a local vegetable market of the Xi'an region of Shaanxi Province, China. Pentafluorobenzyl bromide (PFB-Br) and tetraoctylammonium bromide (TOA-Br) were purchased from Sigma (Sigma–Aldrich GmbH, Sternheim, Germany). Sodium nitrate and sodium nitrite were bought from Cambridge Isotope (Andover, MA, USA). Assay kits of serum total-cholesterol (TC), triglycerides (TG), high-density lipoprotein-cholesterol (HDL-C), low-density lipoprotein-cholesterol (LDL-C), and glucose were the products of Huili of Biotechnology (Changchun, China). The commercially diagnostic kit of NO and nitric oxide synthase (NOS) were obtained from the Jiancheng Institute of Biotechnology (Nanjing, China). The ELISA kits of insulin, F_2_-isoprostanes, interleukin 6 (IL-6), tumor necrosis factor α (TNF-α), C-reactive protein (CRP), and endothelin 1 (ET-1) were obtained from the Lantu Biotech Co. Ltd (Fujian, China). Deionized water was prepared using a Millipore Milli Q-Plus system (Millipore, Bedford, MA). HPLC grade acetonitrile and methanol were purchased from Honeywell (Morristown, NJ, USA). Other chemicals used in the study were of analytic grade and commercially available.

### Sample preparation

To remove the nitrate from spinach, clean fresh spinach was boiled thrice with warm water at 70°C (40 min each time), filtered, and freeze-dried with a lyophilizer. The residue was freeze-dried using a lyophilizer to make the nitrate-removed spinach. To obtain the intact spinach rich in nitrate, clean fresh spinach was directly homogenized and freeze-dried. Finally, the samples were shattered by a disintegrator and saved as a standby (Voucher specimens of the plant materials were deposited at College of Food Engineering and Nutritional Science, Shaanxi Normal University, China).

### Derivatization of $\text{N}{\text{O}}_{2}^{-}$ and $\text{N}{\text{O}}_{3}^{-}$

Aqueous standard solutions (NaNO_2_ and NaNO_3_) or spinach samples (300 µL) were treated with 500 µL of 8.0 mM TOA-Br in acetone and 50 µL of 20% PFB-Br in acetone at 50°C for 30 min (16). Samples were evaporated under N_2_ to remove acetone. The reaction products were extracted from the remaining aqueous phase with toluene (1.0 mL) and then centrifuged at 3,000*g* for 10 min at 4°C. A volume of 200 µL of supernatant was extracted with 1 mL acetonitrile again after removing the toluene. Finally, the organic phase was collected for HPLC analysis (16).

### HPLC quantification of $\text{N}{\text{O}}_{2}^{-}$ and $\text{N}{\text{O}}_{3}^{-}$

The HPLC analysis of nitrite and nitrate was performed on a Shimadzu LC-2010A HPLC system. The analytical column used was a RP-C_18_ column (4.6 mm i.d.×250 mm, 5 µm, Venusil, Wilmington, DE, USA), and the separation was performed at 30°C. A gradient elution was performed by varying the proportion of solvent A (acetonitrile) to solvent B (water). The gradient program was as follows: 0–10 min from 75 to 60% B, 10–30 min from 60 to 40% B, and 30–40 min from 40 to 10% B. Elution was carried out at a flow rate of 1.0 mL/min, and the wavelength for UV detection was 218 nm. The injection volume was 20 µL.

### Animal experiment

In total, 40 healthy male Swiss-Kunming mice (weighing 22±2 g) were purchased from the Experimental Animal Center of the Fourth Military Medical University (Xi'an, China). The mice were housed in cages in a standardization animal laboratory, which was maintained at a temperature of 22–24°C and a humidity of 50–55% under a 12:12 light–dark cycle, with free access to water and rodent chow (40% corn flour, 26% wheat flour, 10% bran, 10% fish meal, 10% bean cake, 2% mineral, 1% coarse, and 1% vitamin complex; Qianmin Feed Factory, Xi'an, China). The experimental animal procedures were in accordance with the Regulations of Experimental Animal Administration of the Fourth Military Medical University Committee on Animal Care and Use (approval number XJYYLL-2015689).

After a 1-week acclimation period, mice were given high-fructose (20% fructose-deionized water) and a high-fat diet (93.8% rodent chow, 1% cholesterol, 5% lard, and 0.2% bile salt complex; Qianmin Feed Factory). Simultaneously, mice were randomly divided into four groups to receive different concentrations of nitrate with eight mice in each group: model group (0 mg/kg $\text{N}{\text{O}}_{3}^{-}$, 0.5 mL of solution consisting of 100% nitrate-removal spinach), low-dose group (15 mg/kg $\text{N}{\text{O}}_{3}^{-}$, 0.5 mL of solution containing 75% nitrate-removed spinach and 25% intact spinach rich in nitrate), medium-dose group (30 mg/kg $\text{N}{\text{O}}_{3}^{-}$, 0.5 mL of solution containing 50% nitrate-removed spinach and 50% intact spinach rich in nitrate), and high-dose group (60 mg/kg $\text{N}{\text{O}}_{3}^{-}$, 0.5 mL of solution consisting of 100% intact spinach rich in nitrate). Mice which were given a normal diet and 0 mg/kg $\text{N}{\text{O}}_{3}^{-}$ were used as the normal group. The powder of spinach dissolved in 5% carboxymethylcellulose sodium (CMC-Na, 0.4 mL, m/v) was administered by oral gavage once daily (9:00 a.m.) for 28 consecutive days. At the end of the experiment, all the animals were euthanized and sacrificed to obtain the blood and livers. The blood was collected by cardiac puncture.

### Ozone-based chemiluminescence assay of $\text{N}{\text{O}}_{2}^{-}$

The plasma nitrite was detected by gas-phase chemiluminescence assay using our previously described method (16, 17). Blood was collected into chilled tubes containing 50 µL of the mixed NEM/EDTA (10/2.0 mM) and immediately mixed and centrifuged at 3,000*g* for 5 min at 4°C. Fresh plasma was kept on ice in the dark and was analyzed within an hour. The tri-iodide solution (2 mL) and glacial acetic acid (6 mL) were delivered into a glass purge vessel with a rubber septum-covered injection inlet and connected to a gas ice-trap cooler with 15 mL of 0.5 M NaOH solution in the outlet. The resulting ${\text{I}}_{3}^{-}$ solution reduces $\text{N}{\text{O}}_{2}^{-}$ to NO gas, which can be detected by a sensitive ozone-based chemiluminescence analyzer (CLD66, Eco Physics, Duernten, Switzerland). The tri-iodide solution was prepared by dissolving 1.0 g KI and 0.4 g I_2_ in 20 mL H_2_O. A volume of 300 µL of the tested samples or nitrate standard solutions was quickly injected into the purge vessel under NO-free N_2_ atmosphere. Fresh plasma was spiked with 10% antifoam before injection. The concentration of nitrite was quantified by the NO signal peak area. All containers were adequately washed with Milli-Q water to remove $\text{N}{\text{O}}_{2}^{-}$ contamination.

### Determination of blood biochemical indexes

The serum levels of TC, TG, HDL-C, LDL-C, GLU, NO, and NOS were assessed with commercially available diagnostic kits, and the results were expressed in mmol/L (for TC, TG, HDL-C, LDL-C, and NO), µmol/L (for GLU), and U/mL (for NOS). The serum concentrations of insulin, F_2_-isoprostanes, IL-6, CRP, TNF-α, and ET-1 were measured using the ELISA kits, and the results were expressed in µU/mL (for insulin), pg/mL (for F_2_-isoprostanes and IL-6), ng/mL (for CRP), and ng/L (for TNF-α and ET-1). The atherogenic index (AI) was calculated by the following formula (18):

AI=(TC-HDL-C)/HDL-C

The homeostasis model assessment-insulin resistance (HOMA-IR) index was computed from the glucose (mmol/L) and insulin (µU/mL) levels:

HOMA-IR=glucose(mmol/L)×insulin(μU/mL)/22.5

### Histopathological examination

Histology of liver was studied using hematoxylin and eosin (H&E) and Oil Red O staining (19, 20). The fresh liver samples were rapidly dissected, and the liver tissue sections (5 mm) were fixed by immersing in a 10% neutral formalin solution at room temperature for 24 h. The fixed tissues were embedded in paraffin, and sectioned (5–6 µm thick) and stained with H&E. For Oil Red O staining, the frozen liver samples were processed using a cryostat and then fixed and stained. The histopathological changes in the sections were observed by a light photomicroscope.

### Statistical analysis

All experiments were performed at least in triplicate, and the results were expressed as mean±SD (standard deviation). Data obtained were analyzed using one-way analysis of variance (ANOVA) and Duncan's multiple range tests. The difference is considered statistically significant if *p<*0.05 (SPSS, version 16.0).

## Results

### The nitrate and nitrite content in spinach

The analytical results of the nitrate and nitrite content in spinach after different processing were showed in Table 1. In fresh spinach, the nitrate content (1,913.1±182.4 mg/kg) was 10,000 times higher than the nitrite content (189.3±72.1 µg/kg). After boiling in water for three times, the contents of nitrate and nitrite in spinach were sharply dropped to 3.1±0.6 mg/kg and 69.4±17.3 µg/kg, respectively. The significant decrease in nitrate levels confirmed that the process of boiling with water combined with further filtration could effectively remove excessive amounts of nitrate from spinach.

**Table 1:** The content of $\text{N}{\text{O}}_{3}^{-}$ and $\text{N}{\text{O}}_{2}^{-}$ in spinach with different processing

Spinach	Fresh^a^	Boiled dried^b^
Nitrate (mg/kg)	1913.1±182.4	3.1±0.6
Nitrite (µg/kg)	189.3±72.1	69.4±17.3

a Clean fresh spinach was directly homogenized and freeze-dried

b clean fresh spinach was boiled with warm water at 70°C for three times (40 min each time), and then the residue was freeze-dried.

### Effect of spinach nitrate ingestion on plasma nitrite, serum ET-1, NO levels, and NOS activity

**Fig. 1:**
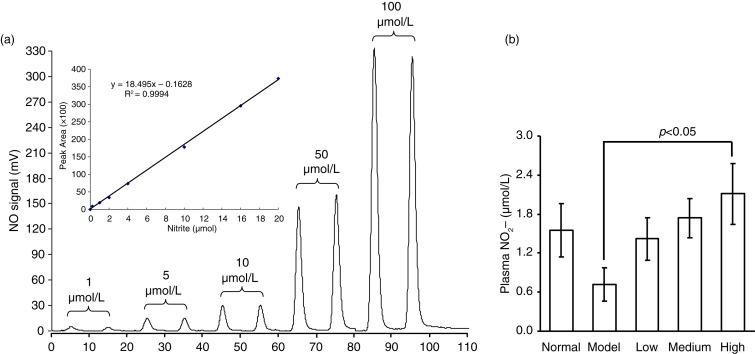
The effect of dietary spinach nitrate on the plasma $\text{N}{\text{O}}_{2}^{-}$ levels. (a) The NO signal intensity of different concentrations of $\text{N}{\text{O}}_{2}^{-}$ and the corresponding standard curve. (b) The plasma $\text{N}{\text{O}}_{2}^{-}$ concentration in mice treated with spinach at different dosages of nitrate. Results are expressed as means±SD of eight mice in each group.

**Fig. 2:**
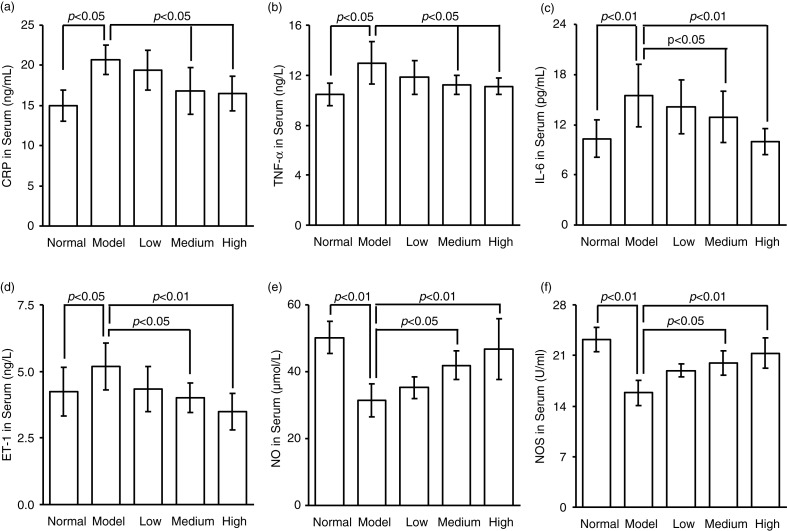
The effect of dietary spinach nitrate on the serum CRP (a), TNF-α (b), IL-6 (c), ET-1 (d), and NO (e) levels and the NOS activity (f) in mice. Data are means±SD, *n*=8.

**Fig. 3:**
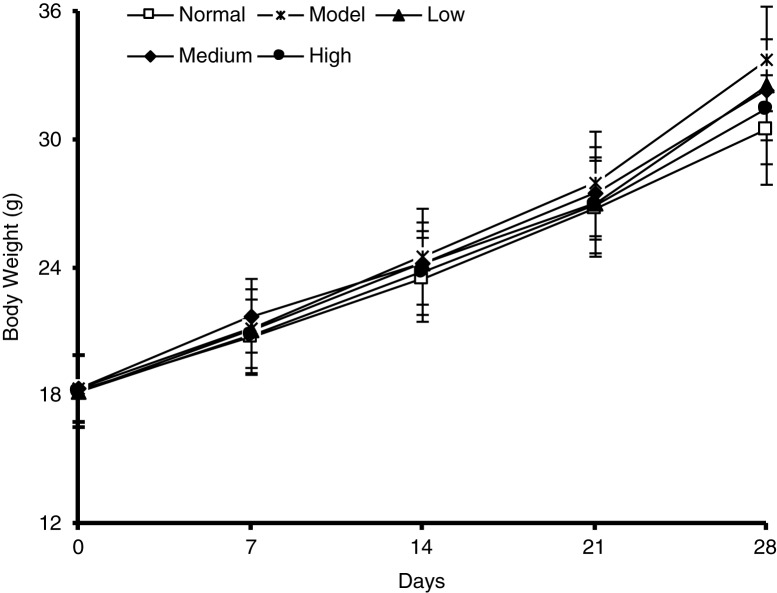
Effect of spinach nitrate on body weight. Results are expressed as means±SD of eight mice in each group.

Figure 1 shows the plasma nitrite levels in mice receiving the processed spinach with different doses of nitrate measured by gas-phase chemiluminescence assay. The chemiluminescence signal area exhibited a strict linear correlation with the amount of $\text{N}{\text{O}}_{2}^{-}$ in a certain range (0–20 µmol), with the linear equation of *Y*=18.495X − 0.1628 (*R*^2^=0.9994) (Fig. 1a). As shown in Fig. 1b, the plasma nitrite content in mice was elevated by the intake of spinach nitrate in a dose-dependent manner, and the mice receiving spinach containing 60 mg/kg of nitrate showed a significant higher level (2.11±0.47 µmol/L) than the mice treated with nitrate-removed spinach (0.72±0.26 µmol/L, *p<*0.05). Furthermore, the influences of dietary spinach nitrate on serum ET-1, NO levels, and NOS activity in mice were shown in Fig. 2d, e, and f, respectively. In comparison with the normal mice, a high-fat and high-fructose diet remarkably elevated the serum ET-1 levels and reduced the NO levels and NOS activity in the model mice. However, both the serum NO level and NOS activities were raised by spinach nitrate ingestion in a dose-dependent manner, where the mice given spinach with 30 and 60 mg/kg of nitrate showed significantly higher NO levels and NOS activities than the model mice, respectively (*p*<0.05 and <0.01). Moreover, the serum ET-1 level in mice decreased after giving nitrate-rich spinach for 28 days, where it dropped from 5.18±0.88 ng/L for the model group to 4.34±0.86 ng/L(*p*>0.05), 4.01±0.57 ng/L (*p<*0.05), and 3.50±0.69 ng/L (*p<*0.01) for the low-, medium-, and high-dose groups, respectively. Notably, no significant differences were observed between the body weight of nitrate-enriched spinach-treated mice and the nitrate-removed model mice (Fig. 3).

### Effects of spinach nitrate on the levels of serum TG, TC, LDL-C, HDL-C, and AI

To determine whether spinach nitrate caused changes of serum lipid profile in mice, the serum levels of TG, TC, LDL-C, and HDL-C were assessed. As depicted in Table 2, the mice in model group exhibited significantly high levels of serum TG, TC, and LDL-C (*p<*0.05, <0.01, and <0.01), and low levels of serum HDL-C (*p<*0.01), in comparison with the normal mice. Spinach nitrate dose-dependently reduced the elevated serum TG, TC, and LDL-C levels of mice with high-fat and high-fructose consumption, and the differences between high-dose group (60 mg/kg) and model group (0 mg/kg) were statistically significant (*p<*0.05). It was noteworthy that all the spinaches containing 15, 30, and 60 mg/kg of nitrate led to a remarkable increase in the serum HDL-C levels, with values of 0.41±0.05, 0.43±0.09, and 0.47±0.07 mmol/L, respectively (*p<*0.05, <0.05, and <0.01), as compared to the model mice (0.30±0.04 mmol/L). Furthermore, AI of high-fat and high-fructose diet-fed mice receiving spinach containing 0, 15, 30, and 60 mg/kg of nitrate was 5.13±1.18, 3.84±0.92, 3.19±1.04, and 2.73±0.76, respectively. The marked drop of AI caused by nitrate-enriched spinach suggested that spinach nitrate had the ability to alleviate hyperlipidemia.

**Table 2:** The effect of dietary intake of spinach nitrate on serum markers in mice

Group	Normal (0 mg/kg)	Model (0 mg/kg)	Low (15 mg/kg)	Medium (30 mg/kg)	High (60 mg/kg)
Serum TG (mmol/L)	2.17±0.39	2.83±0.46#	2.69±0.42	2.51±0.50	2.23±0.41*
Serum TC (mmol/L)	1.61±0.30	2.13±0.41##	1.96±0.45	1.80±0.38	1.68±0.32**
Serum LDL-C (mmol/L)	0.85±0.10	1.16±0.17##	1.09±0.09	0.98±0.09*	0.90±0.09**
Serum HDL-C (mmol/L)	0.49±0.06	0.30±0.04##	0.41±0.05*	0.43±0.09*	0.47±0.07**
Atherogenic index (AI)	2.48±0.71	5.13±1.18##	3.84±0.92*	3.19±1.04*	2.73±0.76**
F_2_-isoprostanes (pg/mL)	14.25±5.91	26.75±7.95##	22.51±8.57	19.64±8.30	16.89±6.76*
Serum GLU (mmol/L)	6.26±1.21	9.12±2.20##	7.97±1.74	7.08±1.63*	6.13±1.86**
Serum INS (µU/mL)	3.27±0.41	5.01±0.55##	4.65±0.46	4.19±0.33*	3.75±0.17**
HOMA-IR	0.91±0.26	2.05±0.58##	1.62±0.33*	1.32±0.37**	1.02±0.34**

Values are expressed as means±SD of eight mice in each group.

# *p* < 0.05

## *p* < 0.01, relative to the normal group.

* *p* < 0.05

** *p* < 0.01, relative to the model group.

### Effects of spinach nitrate on the serum levels of glucose, insulin, and HOMA

The serum glucose and insulin concentrations in mice given spinach with or without nitrate were presented in Table 2. Administration of high-fat and high-fructose diet caused a significant increase in serum glucose levels (*p<*0.01) and insulin levels (*p<*0.01), as compared with the normal mice. After spinach nitrate intervention, both the serum glucose and insulin levels of high-fat and high-fructose diet-fed mice decreased. Spinach with 30 and 60 mg/kg of nitrate caused evident fall in the serum glucose levels from 9.12±2.20 mmol/L of the model group to 7.08±1.63 mmol/L (*p<*0.05) and 6.13±1.86 mmol/L (*p<*0.01), respectively. Similarly, the insulin level sharply declined to 4.19±0.33 µU/mL (*p<*0.05) for the mice given spinach containing 30 mg/kg of nitrate, and 3.75±0.17 µU/mL (*p<*0.01) for the mice given spinach with 60 mg/kg of nitrate, in contrast to the model mice (5.01±0.55 µU/mL). To quantify the insulin sensitivity, HOMA was used. As depicted in Table 2, the HOMA-IR index was tremendously decreased from 2.05±0.58 of the model group (0 mg/kg) to 1.62±0.33, 1.32±0.37, and 1.02±0.34 of the low-dose (15 mg/kg), medium-dose (30 mg/kg), and high-dose (60 mg/kg) groups, respectively (*p<*0.05, <0.01, and <0.01), indicating that spinach nitrate could effectively improve the insulin resistance.

### Effect of spinach nitrate on the serum F_2_-isoprostanes levels

To further evaluate the effect of spinach nitrate on the degree of lipid peroxidation, we examined the serum F_2_-isoprostanes level, which is considered as the gold standard for quantifying lipid peroxidation/oxidative stress *in vivo* (21). High-fat and high-fructose diet caused a remarkable increase in F_2_-isoprostanes levels from 14.25±5.91 pg/mL of normal mice to 26.75±7.95 pg/mL (*p<*0.01). Our results demonstrated that supplementation with nitrate-rich spinach could effectively lower the serum concentration of F_2_-isoprostanes. The serum F_2_-isoprostanes level of the model mice was 26.75±7.95 pg/mL, whereas for the mice given spinach containing 15, 30, and 60 mg/kg of nitrate, the levels decreased to 22.51±8.57, 19.64±8.30, and 16.89±6.76 pg/mL (*p<*0.05), respectively (Table 2), implying a subdued oxidative stress and lipid peroxidation in mice administrated with nitrate-enriched spinach.

### Effects of spinach nitrate on the serum CRP, IL-6, and TNF-α levels

Figure 2a–c illustrates the serum CRP, TNF-α, and IL-6 concentrations of mice supplemented with spinach containing different dosages of nitrate, respectively. High-fat and high-fructose diet significantly elevated the serum levels of CRP, IL-6, and TNF-α in mice. However, it was found that spinach nitrate decreased the elevated serum CRP, TNF-α, and IL-6 levels in dose-dependent manners. More specifically, the CRP levels of mice treated with spinach containing 30 and 60 mg/kg of nitrate (16.79±2.94 and 16.50±2.13 pg/mL, *p<*0.05 and <0.05, respectively) were significantly lower than that of the model mice (20.68±1.86 pg/mL), but the decrease caused by spinach with 15 mg/kg of nitrate was not distinct (*p>*0.05). Similar trends were observed in the serum TNF-α and IL-6 levels after spinach nitrate intervention.

### Histopathological examination of mouse liver

**Fig. 4:**
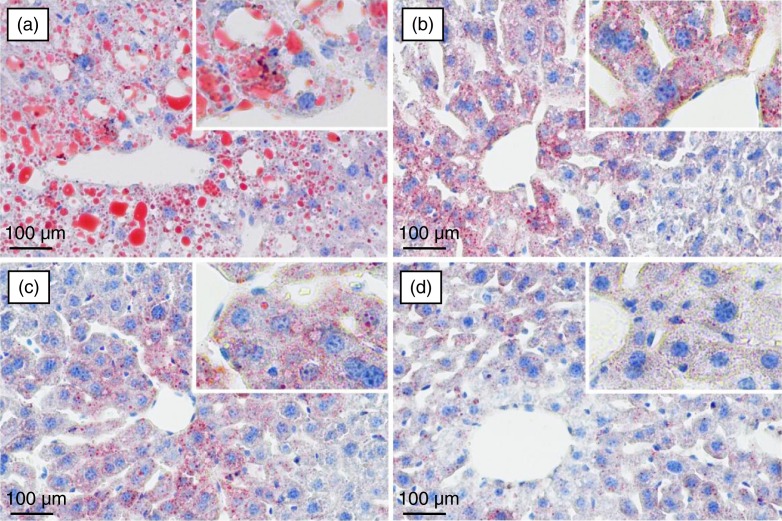
Lipid staining of the liver sections from high-fat and high-fructose treated mice using Oil Red O staining: (a) model group (0 mg/kg), (b) low-dose group (15 mg/kg), (c) medium-dose group (30 mg/kg), and (d) high-dose group (60 mg/kg).

**Fig. 5:**
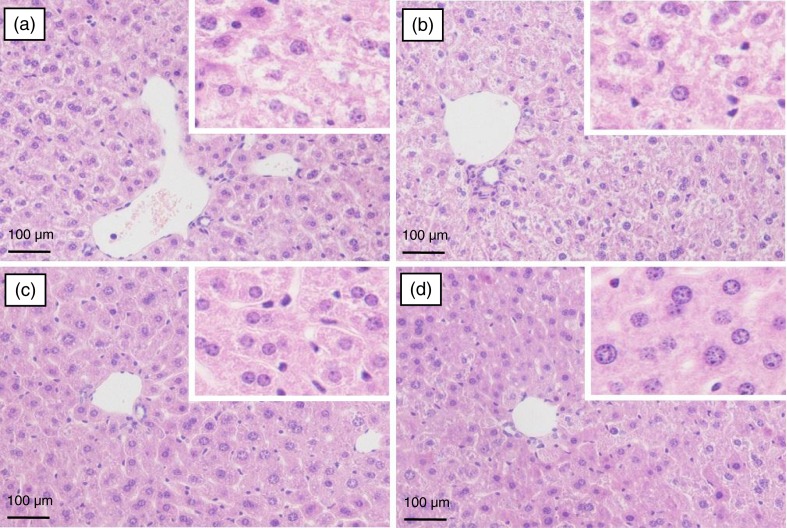
The effect of spinach nitrate on histopathological changes of liver hepatocytes stained with H&E in high-fat and high-fructose fed mice: (a) model group (0 mg/kg), (b) low-dose group (15 mg/kg), (c) medium-dose group (30 mg/kg), and (d) high-dose group (60 mg/kg).

The histopathological observation of mouse liver was performed to further support the results of the serum biochemical analysis. Representative photomicrographs of liver tissues with Oil Red O and H&E staining are shown in Figs. 4 and 5, respectively. Significant lipid accumulation was observed in the liver tissues of the model mice (Fig. 4a), whereas the hepatic fat deposit induced by the high-fat and high-fructose diet was evidently ameliorated after spinach nitrate intervention (Fig. 4b–d). The spinach with 60 mg/kg of nitrate exhibited a much higher efficacy than spinach containing with 15 and 30 mg/kg of nitrate. The H&E-stained liver specimen showed that the acute administration of the high-fat and high-fructose diet led to severe cellular degeneration, hepatocyte necrosis, and cytoplasmic vacuolation in mice (Fig. 5a). However, the hepatic histopathological changes were apparently improved by the treatment with nitrate-rich spinach, and the mice treated with spinach containing 60 mg/kg of nitrate showed well-preserved cytoplasm, prominent nuclei, and legible nucleoli (Fig. 5b–d). The results of the histopathological examination well supported the consequences of serum biochemical markers and lipid peroxidation.

## Discussion

Dietary inorganic nitrate ($\text{N}{\text{O}}_{3}^{-}$) and nitrite ($\text{N}{\text{O}}_{2}^{-}$) have been considered to be toxic constituents in our diet for half a century because of their proposed association with the development of gastric cancer and other malignancies, and the concentrations of them are strictly limited in food and drinking water (22, 23). However, scientific and medical interest has grown exponentially in the beneficial effects of dietary or inorganic nitrate on cardiovascular health in recent years (24). NO was shown to modulate insulin sensitivity, and a decreased bioavailability of NO contributes to endothelial dysfunction (25). Lundberg et al. reported that dietary inorganic nitrate exerted robust NO-like effects in our human body including lowering blood pressure, inhibiting platelet aggregation, preventing endothelial dysfunction, and protecting vascular function (22). Diet is a major source of nitrate, and the content of naturally occurring nitrate is particularly high in certain green leafy vegetables, including spinach, rocket, lettuce, beetroot, and Chinese cabbage (26). Therefore, this study was designed to elucidate the influences of dietary nitrate on insulin resistance, endothelial dysfunction, and inflammation in mice. To achieve this goal, spinach as a source of dietary nitrate was administered to mice for 28 days. As a result, the nitrate-rich spinach positively regulated glucose and lipid metabolism, insulin resistance, inflammation, and endothelial dysfunction markers in mice.

Spinach, a common green leafy vegetable, has been reported to have a high content of nitrate (27). Dietary nitrate is mostly transformed into nitrite by oral symbiotic bacterial nitrate reductases and then converted into NO in the gastrointestinal tract and vascular smooth muscle cells under the action of key enzymes including nitrite reductase, xanthine oxidoreductase, aldehyde oxidase, deoxidized hemoglobin, myoglobin, and cytochrome *c* oxidase, thereby exhibiting regulative effects on endothelial function in the body (28). In this study, to illustrate the metabolism of dietary nitrate *in vivo*, we determined the plasma nitrite content using ozone-based chemiluminescence assay. It was found that the nitrite concentration in plasma was dose-dependently increased by administration of spinach with different doses of nitrate, indicating that the nitrate from spinach was metabolized to nitrite in the body of mice. In addition, no significant differences were found in the body weight between nitrate-rich spinach treated mice and model mice (Fig. 3), suggesting the non-toxic property of spinach nitrate at the dose of 60 mg/kg on mice.

Emerging evidences have showed that a combined use of a high-fat and high-fructose diet may predispose to insulin resistance in animals, such as abdominal fat deposition, glucose tolerance, dyslipidemia, hyperinsulinemia, and hypertension (29–31). In recent years, high-fructose consumption has been confirmed to effectively cause insulin resistance, hypertriglyceridemia, hepatic steatosis, non-alcoholic fatty liver, hypertension, and metabolic disorders, as well as vascular lesions (18, 32). High fat feeding was also considered as a good manner for hyperlipidemia animal model establishment (33). Furthermore, the high-fat and high-fructose diet increases the excessive reactive oxygen species (ROS) generated by the renin–Ang II system and mitochondrial dysfunction (34). ROS generation mediates a pro-inflammatory cascade, which can cause an increase of adipogenesis, release of inflammatory cytokines, and decrease in adiponectin, leading to insulin resistance and vascular endothelial dysfunction (34, 35). In this study, spinach samples with different concentrations of nitrate were given intragastrically to the mice fed with high-fructose (20%, w/v) and high-fat (1% cholesterol and 5% lard, w/w) diet for 28 consecutive days. Our results showed that the high-fat and high-fructose diet induced significant reduction of serum HDL-C level and elevation of serum TG, TC, LDL-C, glucose, and insulin levels in mice (Table 2). However, ingestion of spinach nitrate significantly lowered the elevation of TG, TC, LDL-C, glucose, and insulin levels, and remarkably enhanced the decrease of HDL-C level in the mice fed with the high-fat and high-fructose diet. Furthermore, the calculated AI and HOMA-IR values of nitrate-enriched spinach-treated mice were significantly lower than that of the model mice, suggesting that spinach nitrate effectively improved the impairment of lipid and glucose metabolism, as well as the insulin resistance of mice receiving the high-fat and high-fructose diet. The improvement effect of spinach nitrate on lipid homeostasis was further confirmed by the results of the histopathological observation, where treatment of spinach rich in nitrate effectively ameliorated the hepatic fat deposit, severe cellular degeneration, hepatocyte necrosis, and cytoplasmic vacuolation triggered by the high-fat and high-fructose diet (Fig. 4). F_2_-isoprostanes, which are formed by free radical-catalyzed peroxidation of phospholipid-bound arachidonic acid *in vivo* and *in vitro*, are considered as the most reliable markers of oxidative stress and lipid peroxidation (21, 36). In our hands, it was found that nitrate-enriched spinach obviously inhibited the elevation of serum F_2_-isoprostanes induced by the high-fat and high-fructose diet, indicating the improving effect of spinach nitrate on oxidative stress and lipid peroxidation in mice (Table 2).

CRP, a stable downstream marker of the inflammatory process, has been reported to be an independent predictor of the risk of atherosclerosis, cardiovascular events, and myocardial infarction (26). In addition, IL-6 and TNF-α can play a pivotal role in inflammation-associated disturbances in glucose and insulin metabolism, lipid homeostasis, endothelial dysfunction, and accelerated atherosclerosis (37–39). Herein, our results revealed that the administration of nitrate-enriched spinach resulted in the decreases in serum CRP, IL-6, and TNF-α levels, which were evidently elevated by a high-fructose and high-fat diet in mice (Fig. 2). It is well known that the increasing production of inflammatory cytokines can contribute to endothelial dysfunction (4). IL-6 and TNF-α, which can be elevated in obesity and insulin resistance, have been confirmed to inhibit endothelial-dependent vasodilatation (40), and the degenerative endothelial vasodilatation in response to IL-6 and TNF-α can result in the reduction of NO availability (41). NO is an important protective molecule in the vasculature, and a defect in the production or activity of NO leads to endothelial dysfunction (42), whereas ET-1 is a peptide secreted mostly from vascular endothelial cells with profound vasoconstriction, pro-inflammatory action, stimulation of free radical formation, and platelet activation (43). As a result, ET-1 and NO are natural counterparts in vascular function, and an imbalance between these two mediators is the characteristic of endothelial dysfunction (42). Herein, it was found that the high-fat and high-fructose diet induced a significant increase in serum ET-1 level and a decrease in serum NO level in mice, suggesting a certain degree of endothelial injury. However, supplementation with spinach nitrate remarkably lowered serum ET-1 level and simultaneously raised serum NO level in mice with vascular endothelial injury, suggesting the potential vascular endothelial protective efficacy of spinach nitrate. Moreover, serum NOS activity was dose-dependently enhanced by spinach nitrate in mice. These results suggested that the nitrate-enriched spinach could stimulate the vascular endothelial cell to produce NO via the upregulation of NOS activity. Taken together, this finding indicates that the improvement of vascular NO status by nitrate-enriched spinach may play an important role in remittance of insulin resistance. However, the specific mechanisms underlying these effects need to be explored in the future.

## Conclusions

The present study for the first time restores the prototype of spinach food and establishes a diet intervention with four nitrate content-matched treatments (spinach with or without low, medium, and high dose of nitrate) in high-fat and high-fructose treated mice, highlighting the significant differences in the contents of the nitrate ingredient to be evaluated. This study for the first time showed the ameliorative effects of spinach nitrate on lipid metabolism, insulin resistance, endothelial function, and inflammation in high-fat and high-fructose diet-fed mice. Our findings also suggest that spinach is a promising nitrate resource of dietary supplements for insulin resistance prevention, and provide basic information for exploring the potential benefits of dietary spinach against chronic diseases induced by high fat or high fructose.

## References

[1] Brown AE, Walker M (2016). Genetics of insulin resistance and the metabolic syndrome. Curr Cardiol Rep.

[2] Prasad DS, Kabir Z, Dash AK, Das BC (2012). Prevalence and risk factors for metabolic syndrome in Asian Indians: a community study from urban eastern India. J Cardiovasc Dis Res.

[3] Ford ES, Li C, Zhao G, Pearson WS, Mokdad AH (2008). Prevalence of the metabolic syndrome among US adolescents using the definition from the International Diabetes Federation. Diabetes Care.

[4] Cersosimo E, DeFronzo RA (2006). Insulin resistance and endothelial dysfunction: the road map to cardiovascular diseases. Diabetes Metab Res Rev.

[5] Dandona P, Aljada A, Chaudhuri A, Mohanty P, Garg R (2005). Metabolic syndrome: a comprehensive perspective based on interactions between obesity, diabetes, and inflammation. Circulation.

[6] Steinberg HO, Chaker H, Leaming R, Johnson A, Brechtel G, Baron AD (1996). Obesity/insulin resistance is associated with endothelial dysfunction. Implications for the syndrome of insulin resistance. J Clin Invest.

[7] Sola S, Mir MQ, Cheema FA, Khan-Merchant N, Menon RG, Parthasarathy S (2005). Irbesartan and lipoic acid improve endothelial function and reduce markers of inflammation in the metabolic syndrome: results of the irbesartan and lipoic acid in endothelial dysfunction (ISLAND) study. Circulation.

[8] Lundberg JO, Feelisch M, Björne H, Jansson EA, Weitzberg E (2006). Cardioprotective effects of vegetables: is nitrate the answer?. Nitric Oxide.

[9] Machha A, Schechter AN (2012). Inorganic nitrate: a major player in the cardiovascular health benefits of vegetables?. Nutr Rev.

[10] Kapil V, Milsom AB, Okorie M, Maleki-Toyserkani S, Akram F, Rehman F (2010). Inorganic nitrate supplementation lowers blood pressure in humans: role for nitrite-derived NO. Hypertension.

[11] Bryan NS, Calvert JW, Elrod JW, Gundewar S, Ji SY, Lefer DJ (2007). Dietary nitrite supplementation protects against myocardial ischemia-reperfusion injury. Proc Natl Acad Sci USA.

[12] Carlström M, Larsen FJ, Nyström T, Hezel M, Borniquel S, Weitzberg E (2010). Dietary inorganic nitrate reverses features of metabolic syndrome in endothelial nitric oxide synthase-deficient mice. Proc Natl Acad Sci USA.

[13] Gilchrist M, Shore AC, Benjamin N (2011). Inorganic nitrate and nitrite and control of blood pressure. Cardiovasc Res.

[14] Hord NG, Tang Y, Bryan NS (2009). Food sources of nitrates and nitrites: the physiologic context for potential health benefits. Am J Clin Nutr.

[15] Santamaria P (2006). Nitrate in vegetables: toxicity, content, intake and EC regulation. J Sci Food Agric.

[16] Yang X, Bondonno CP, Indrawan A, Hodgson JM, Croft KD (2013). An improved mass spectrometry-based measurement of NO metabolites in biological fluids. Free Radic Biol Med.

[17] Bondonno CP, Yang X, Croft KD, Considine MJ, Ward NC, Rich L (2012). Flavonoid-rich apples and nitrate-rich spinach augment nitric oxide status and improve endothelial function in healthy men and women: a randomized controlled trial. Free Radic Biol Med.

[18] Makni M, Fetoui H, Garouiel M, Gargouri NK, Jaber H, Makni J (2010). Hypolipidemic and hepatoprotective seeds mixture diet rich in ω-3 and ω-6 fatty acids. Food Chem Toxicol.

[19] Tappy L, Lê KA, Tran C, Paquot N (2010). Fructose and metabolic diseases: new findings, new questions. Nutrition.

[20] Jaya C, Anuradha CV (2010). *Cissus quadrangularis* stem alleviates insulin resistance, oxidative injury and fatty disease in rats fed high fat plus fructose diet. Food Chem Toxicol.

[21] Comporti M, Signorini C, Arezzini B, Vecchio D, Monaco B, Gardi C (2008). F_2_-isoprostanes are not just markers of oxidative stress. Free Radic Biol Med.

[22] Lundberg JO, Carlström M, Larsen FJ, Weitzberg E (2011). Roles of dietary inorganic nitrate in cardiovascular health and disease. Cardiovasc Res.

[23] Mirvish SS (1995). Role of N-nitroso compounds (NOC) and N-nitrosation in etiology of gastric, esophageal, nasopharyngeal and bladder cancer and contribution to cancer of known exposures to NOC. Cancer Lett.

[24] Kevil CG, Lefer DJ (2011). Review focus on inorganic nitrite and nitrate in cardiovascular health and disease. Cardiovasc Res.

[25] Sydow K, Mondon CE, Cooke JP (2005). Insulin resistance: potential role of the endogenous nitric oxide synthase inhibitor ADMA. Vasc Med.

[26] Lidder S, Webb AJ (2012). Vascular effects of dietary nitrate (as found in green leafy vegetables and beetroot) via the nitrate–nitrite–nitric oxide pathway. Br J Clin Pharmacol.

[27] 
Correia M, Barroso Â, Barroso MF, Soares D, Oliveirab M, Delerue-Matos C (2010). Contribution of different vegetable types to exogenous nitrate and nitrite exposure. Food Chem.

[28] Lundberg JO, Weitzberg E, Gladwin MT (2008). The nitrate-nitrite-nitric oxide pathway in physiology and therapeutics. Nat Rev Drug Discov.

[29] Zhang ZH, Wang ZQ, Yang Z, Niu YX, Zhang WW, Li XY (2015). A novel mice model of metabolic syndrome: the high-fat–high-fructose diet-fed ICR mice. Exp Anim.

[30] Poudyal H, Panchal S, Brown L (2010). Comparison of purple carrot juice and β-carotene in a high-carbohydrate, high-fat diet-fed rat model of the metabolic syndrome. Br J Nutr.

[31] Wada T, Kenmochi H, Miyashita Y, Sasaki M, Ojima M, Sasahara M (2010). Spironolactone improves glucose and lipid metabolism by ameliorating hepatic steatosis and inflammation and suppressing enhanced gluconeogenesis induced by high-fat and high-fructose diet. Endocrinology.

[32] Miatello R, Vázquez M, Renna N, Cruzado M, Zumino AP, Risler N (2005). Chronic administration of resveratrol prevents biochemical cardiovascular changes in fructose-fed rats. Am J Hypertens.

[33] Axelsen LN, Pedersen HD, Petersen JS, Holstein-Rathlou NH, Kjølbye AL (2010). Metabolic and cardiac changes in high cholesterol–fructose-fed rats. J Pharmacol Toxicol Methods.

[34] Chidambaram J, Carani VA (2010). *Cissus quadrangularis* stem alleviates insulin resistance, oxidative injury and fatty liver disease in rats fed high fat plus fructose diet. Food Chem Toxicol.

[35] Lehnen AM, Rodrigues B, Irigoyen MC, De Angelis K, Schaan BD (2013). Cardiovascular changes in animal models of metabolic syndrome. J Diabetes Res.

[36] Stoner L, Lucero AA, Palmer BR, Jones LM, Young JM, Faulkner J (2013). Inflammatory biomarkers for predicting cardiovascular disease. Clin Biochem.

[37] Patterson CC, Smith AE, Yarnell JW, Rumley A, Ben-Shlomo Y, Lowe GD (2010). The associations of interleukin-6 (IL-6) and downstream inflammatory markers with risk of cardiovascular disease: the Caerphilly Study. Atherosclerosis.

[38] Nishida H, Horio T, Suzuki Y, Iwashima Y, Tokudome T, Yoshihara F (2012). Interleukin-6 as an independent predictor of future cardiovascular events in high-risk Japanese patients: comparison with C-reactive protein. Cytokine.

[39] Al-Aly Z, Pan H, Zeringue A, Xian H, McDonald JR, El-Achkar TM (2011). Tumor necrosis factor-α blockade, cardiovascular outcomes, and survival in rheumatoid arthritis. Transl Res.

[40] Diamant M, Lamb HJ, van de Ree MA, Endert EL, Groeneveld Y, Bots ML (2005). The association between abdominal visceral fat and carotid stiffness is mediated by circulating inflammatory markers in uncomplicated type 2 diabetes. J Clin Endocrinol Metab.

[41] Ritchie SA, Connell JM (2007). The link between abdominal obesity, metabolic syndrome and cardiovascular disease. Nutr Metab Cardiovasc Dis.

[42] Bourque SL, Davidge ST, Adams MA (2011). The interaction between endothelin-1 and nitric oxide in the vasculature: new perspectives. Am J Physiol Regul Integr Comp Physiol.

[43] Böhm F, Pernow J (2007). The importance of endothelin-1 for vascular dysfunction in cardiovascular disease. Cardiovasc Res.

